# Immune cell-specific transcriptional profiling highlights distinct molecular pathways controlled by Tob1 upon experimental autoimmune encephalomyelitis

**DOI:** 10.1038/srep31603

**Published:** 2016-08-22

**Authors:** Alessandro Didonna, Egle Cekanaviciute, Jorge R. Oksenberg, Sergio E. Baranzini

**Affiliations:** 1 Department of Neurology, University of California San Francisco, San Francisco, California 94158, USA

## Abstract

Multiple sclerosis (MS) is an autoimmune disease of the central nervous system characterized by focal lymphocytic infiltration, demyelination and neurodegeneration. Despite the recent advances in understanding MS molecular basis, no reliable biomarkers have been identified yet to monitor disease progression. Our group has previously reported that low levels of TOB1 in CD4^+^ T cells are strongly associated with a higher risk of MS conversion in individuals experiencing an initial demyelinating event. Consistently, Tob1 ablation in mice exacerbates the clinical phenotype of the MS model experimental autoimmune encephalomyelitis (EAE). To shed light on Tob1 molecular functions in the immune system, we have conducted the first cell-based transcriptomic analysis in Tob1^−/−^ and wildtype mice upon EAE. Next-generation sequencing was employed to characterize the changes in gene expression in T and B cells at pre- and post-symptomatic EAE stages. Remarkably, we found only modest overlap among the different genetic signatures, suggesting that Tob1 may control distinct genetic programs in the different cytotypes. This hypothesis was corroborated by gene ontology and global interactome analyses, which highlighted specific cellular pathways in each cellular subset before and after EAE induction. In summary, our work pinpoints a multifaceted activity of Tob1 in both homeostasis and disease progression.

Transducer of ERBB.2-1 (*Tob1*) belongs to the evolutionary conserved Tob/BTG protein family of genes, which includes six members in vertebrates^1^. All proteins from this family exert their anti-proliferative functions through a common N-terminal domain that also regulates their translocation between the cytosolic and nuclear compartments^2^. Tob1 is ubiquitously expressed at different developmental stages, where it participates in a variety of biological processes^3^,^4^. Moreover, Tob1 plays a central role in the immune system to maintain cell quiescence. Specifically, it has been shown to be highly expressed in unstimulated peripheral blood T lymphocytes and undergoing down-regulation during activation^5^.

From a molecular standpoint, Tob1 inhibits cell proliferation at different levels. Tob1 forms a complex with Smad proteins and blocks transcription of mitogenic genes such as cytokines and cyclins by binding to their promoter regions^5^,^6^. In addition to a direct control of transcription, Tob1 also inhibits the expression of mitogenic genes at the level of translation by preventing circularization of the messenger RNA (mRNA) and hence entry into the ribosomal 60S subunit at the 5′ end of the mRNA target^7^. Finally, Tob1 affects mRNA stability by promoting its decay through deadenylation of the poly (A) tail^6^.

We have recently identified Tob1 as a robust biomarker for multiple sclerosis (MS) progression. MS is an autoimmune disease of the central nervous system (CNS) characterized by focal lymphocytic infiltration, microglial activation, demyelination and axonal degeneration^8^. MS initial manifestation consists in an isolated demyelinating event referred as clinically isolated syndrome (CIS). However, not all individuals with CIS develop a clinically definite form of MS^9^. We found that low levels of Tob1 mRNA in CD4^+^ T cells of CIS patients are strongly correlated with a higher risk of early conversion to MS^10^. Subsequent studies employing Tob1-knockout mice confirmed that the absence of Tob1 in the experimental autoimmune encephalomyelitis (EAE) paradigm was associated with increased inflammation, infiltrating T cells and myelin-reactive Th1 and Th17 cells^11^.

Given the physiological role of Tob1 in modulating gene expression at multiple levels, here we further dissect its contribution to autoimmune demyelination by analyzing the molecular pathways controlled by Tob1 in the immune system upon EAE induction. We demonstrate that cell-specific next-generation RNA sequencing is a reliable approach to gain insight into the functional role of Tob1 in the principal immune cytotypes. For the first time, we show that distinct molecular pathways are activated by Tob1 in different cell populations, suggesting a multifaceted activity of Tob1 in both homeostasis and disease progression.

## Results

### Microarray analysis of gene expression in splenocytes from Tob1^−/−^ mice

In order to investigate the transcriptional role of Tob1 in the immune system, we studied the effects of Tob1 absence on the transcriptome of splenocytes upon EAE, a murine model that recapitulates several features of MS^12^. Mononuclear cells (MNCs) were isolated from the spleens of Tob1^−/−^ mice and Tob1^+/+^ littermates at baseline and at day 15 after EAE induction (15 dpi), corresponding to the peak of the disease^11^. Total RNA was purified from each cell sample and subjected to microarray hybridization as described in the Material and Methods section. To better isolate the molecular events genuinely driven by Tob1 ablation, we decided to focus our investigation to cross-genotype comparisons at the selected time points. Differential expression analysis between Tob1^−/−^ and Tob1^+/+^ (wild type) genotypes identified 106 significant genes at baseline and 113 genes at 15 dpi with P < 0.01 (Supplementary Table S1). Minimal overlap exists between the two sets of transcriptional signatures. Indeed, only *Tob1* itself and chromodomain helicase DNA binding protein 7 (*Chd7*) were differentially expressed before and after EAE induction. Interestingly, *Tob1* levels were increased in knockout mice. This apparent paradox is explained by the fact that the disruption of *Tob1* open reading frame does not prevent its transcription but only the translation process, as confirmed by Western blot (Supplementary Fig. S1). We then performed gene ontology (GO) analysis on the differentially expressed genes to look for possible enrichment in GO categories functionally connected with Tob1 activities. No significant enrichment of specific biological processes was found at any of the two selected time points. The presence of multiple cell types in our samples most likely accounts for the negative results of pathway analysis. Thus, we proceeded to a cell-based approach in order to minimize the confounding effects of cellular heterogeneity.

### RNA-seq on immune cell subpopulations

**Figure 1:**
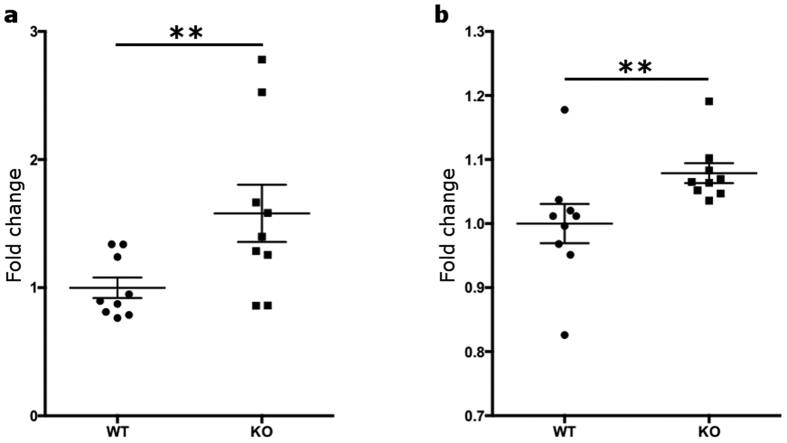
Tob1 ablation increases B cell proliferative capacity. WT or Tob1-KO splenocytes were stimulated with 5 μg/mL lipopolysaccharides (LPS) for 3 days or left unstimulated. Afterwards, cells were fixed and the B cell population immunostained with a PE-conjugated anti-B220. Cell proliferation analysis was then performed by flow cytometry as described in the Materials and Methods section. Tob1-KO B cells show a statistically significant increase in cell proliferation either (**a**) before stimulation or (**b**) after stimulation as compared to WT cells. Results are shown as mean ± SE and derive from three independent experiments, each one using 3 mice per genotype. **P ≤ 0.01, one-tailed T test.

**Figure 2:**
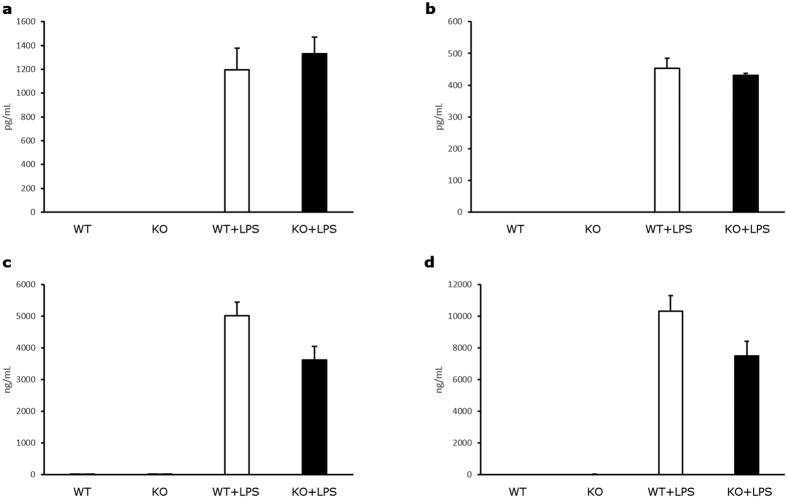
Cytokine and immunoglobulin profiling in Tob1-KO and WT B cells. B cells were isolated by negative selection from the spleens of Tob1 KO or WT mice and then stimulated with 5 μg/mL lipopolysaccharides (LPS) for 3 days or left unstimulated. Conditioned media were subsequently collected and probed for the levels of (**a**) interleukin 6 (Il-6), (**b**) Il-10, (**c**) IgG and (**d**) IgM. No significant differences were detected in the secretion of these molecules between WT and KO cells, before or after LPS stimulation. Results are shown as mean ± SE and derive from at least 3 animals per genotype.

Tob1 is a well-established inhibitor of either CD4^+^ or CD8^+^ T cell proliferation^5^,^11^. However, no evidence exists for an analogous role of Tob1 on B cell proliferative phenotype. For this reason, splenocytes were isolated from Tob1^−/−^ mice and wild type littermates and were stimulated *in vitro* with lipopolysaccharide (LPS). After 3 days, proliferation of B cells was analyzed by FACS using B220 as a marker. Remarkably, Tob1-knockout B cells showed a statistically significant increase in cell division compared to wild type cells either before (one-tailed T-test, P = 0.013) or after stimulation (one-tailed T-test, P = 0.017) (Fig. 1a,b). Furthermore, we tested whether Tob1 deficiency affected the secretion of key cytokines and immunoglobulins (Ig). Untouched B cells isolated by negative selection with a purity >90% (Supplementary Fig. S2) were used for this set of experiments. In details, the concentrations of Il-6 and Il-10 as well as IgG and IgM were measured in the conditioned media from unstimulated and LPS-stimulated B cells. No differences were found between knockout and wild type cells for all the tested factors (Fig. 2a–d.) However, a negative trend was detected for IgG levels in Tob1-knockout B cells with borderline significance (two-tailed T-test, P = 0.068) (Fig. 2c). Thus, we decided to include also the B lineage in the panel of cell subpopulations to test in our transcriptomic analysis.

CD4^+^ T cell, CD8^+^ T cell and B cells were immunopurified by magnetic bead technology from the spleens of Tob1^−/−^ mice and wild type littermates. The purity of the different populations (at least 95%) was further confirmed by qRT-PCR, analyzing the levels of *CD4*, *CD8* and *B220* transcripts in each cell subset (Supplementary Fig. S3). In addition to the disease peak (15 dpi), we extended our analysis to include a pre-symptomatic stage (5 dpi) to better characterize EAE progression. So each cytotype was longitudinally represented by 3 genetic profiles, for a total of 18 datasets between the two mouse genotypes. This time, RNA-seq was employed as sequencing method instead of microarrays.

**Figure 3:**
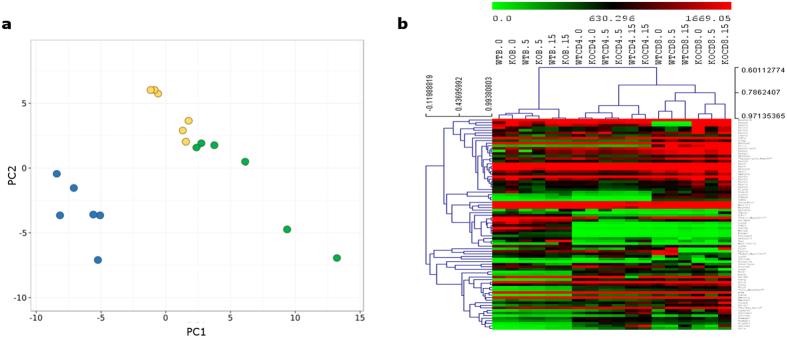
Clustering analysis of immune cell genetic signatures from Tob1-KO and WT mice upon EAE. (**a**) Plot of the first 2 principal components (PC1 on the x-axis and PC2 on the y-axis) calculated from the expression values of the genes with the highest variance that are expressed in CD4^+^ T cells (in yellow), CD8^+^ T cells (in green) and B cells (in blue) isolated from Tob1-deficient and wildtype mice at different EAE stages (0, 5 and 15 dpi). Two main clusters can be recognized, corresponding to B and T cell lineages. (**b**) Unsupervised hierarchical clustering of expression values for the same genes and samples as in panel a. Each row represents a gene while each column corresponds to a different sample. The distances between genes or samples are calculated using Spearman correlation. The different datasets cluster first by cytotype, forming three main groups that correspond to CD4^+^, CD8^+^ and B cell populations. Within each cluster, the influence of disease course prevails on genotype for CD4^+^ T cells and B cells while the opposite trend is observed for CD8^+^ T cells.

In order to decouple the global effects of Tob1 deficiency and EAE progression on the different cell populations, the genes expressed in all the experimental conditions with the highest variance (SD > 150 across all the datasets) were selected from each profile and subjected to principal component analysis (PCA) (Fig. 3a). Principal components 1 and 2 clearly segregated the samples into two distinct clusters –corresponding to the B and T cell lineages– but the contributions of genotype and disease progression were not clearly discriminated. In order to further explore sample aggregation by their expression profiles, we performed unsupervised hierarchical clustering (Fig. 3b). Consistent with the PCA results, the heatmap shows that samples segregate first by cytotype, forming three big clusters. However, the effects of EAE induction and Tob1 ablation were different within each cell population. For example, the genetic signatures of CD4^+^ and B cells cluster first by day of disease and then by genotype. On the contrary, CD8^+^ T cell genetic profiles cluster by genotype first. This effect seems to be driven at least in part by two genes encoding for ribosomal proteins − 60S acidic ribosomal protein P1 (*Rplp1*) and 60S ribosomal protein L41 (*Rpl41*) – which were strongly down-regulated in Tob1^−/−^ CD8^+^ T cells only, irrespectively of the disease stage.

**Figure 4:**
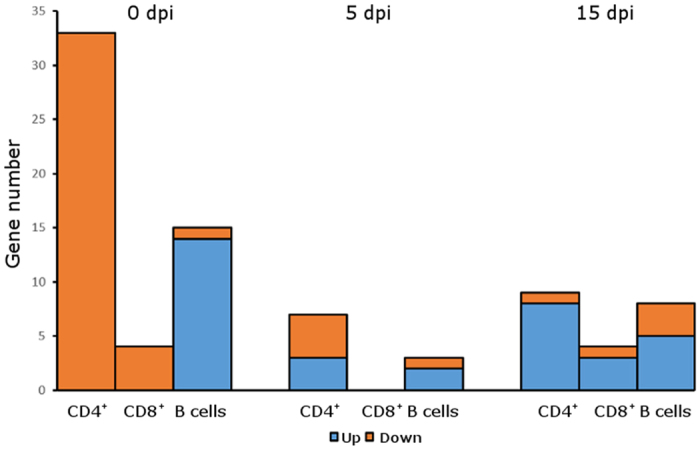
T and B cells respond differently to Tob1 ablation in terms of differentially expressed genes. The number of significant genes that were up- (in blue) or down-regulated (in red) in Tob1-knockout mice compared to wildtype animals was plotted for each cell population at the different EAE stages. At baseline, CD4^+^ and CD8^+^ T cells are characterized by only down-regulated genes while B cells show the opposite tendency with most of the significant genes being upregulated. In contrast, at 5 and 15 dpi, the majority of significant genes is upregulated for the three cytotypes.

Next, we quantified the differences in gene expression between Tob1-knockout and wild type cells for every EAE time point investigated. For each cell type, we plotted the fraction of genes that were significantly up-regulated or down-regulated in Tob1^−/−^ compared to Tob^+/+^ cells (Fig. 4). Notably, at baseline both CD4^+^ and CD8^+^ T cell populations show only down-regulated genes while the vast majority of differentially expressed genes in B cells is up-regulated in Tob1^−/−^ mice. This finding might reflect the molecular commonalities between the two T cell lineages compared to B cells. Interestingly, after EAE induction the differences between T and B cells were strongly mitigated as the ratio between up- and down-regulated genes is closer to 1.

**Figure 5:**
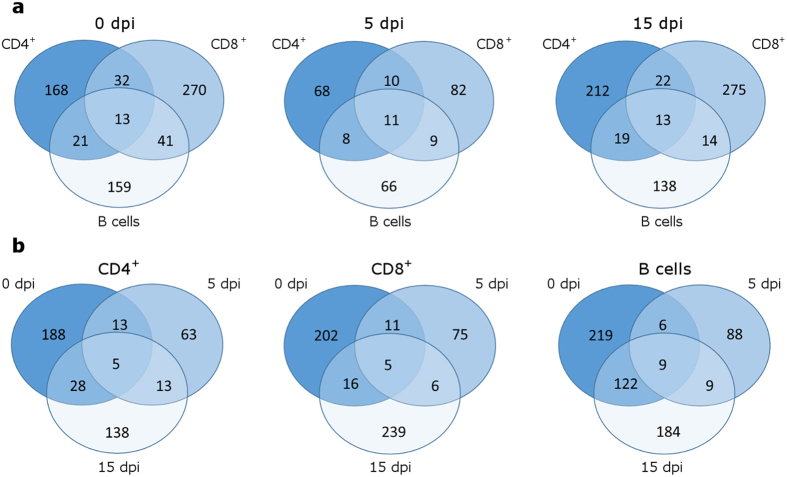
Limited overlap exists among CD4^+^, CD8^+^ and B cell molecular signatures. (**a**) Venn diagrams showing the overlap among the nominally significant genes (Tob1-KO vs WT) of the three immune cell populations at 0, 5 and 15 dpi. (**b**) Venn diagrams showing the overlap among the nominally significant genes identified at the three time points analyzed for each immune cell population. In both cases, the number of genes shared by different cytotypes or disease stages is limited.

We then checked the degree of overlap of differentially expressed genes among the 3 cell subpopulations. Consistently with the results from microarray analysis, almost no overlap exists among significant genes. Thus, we extended our analysis to nominally significant genes but very little overlap was again highlighted at the 3 time points investigated, confirming that most of the genes regulated by Tob1 are cell-specific (Fig. 5a). Only the marginal zone B and B1 cell-specific protein (*Mzb1*) gene was found to be up-regulated in all the 3 Tob1-knockout cytotypes at both baseline and 15 dpi. Notably, also within the same cell subpopulation, the number of nominally significant genes shared at different time points is minimal (Fig. 5b). Only the radical S-adenosyl methionine domain containing 1 (*Rsad1*) gene was found down-regulated at all the time points in both Tob1-knockout CD4^+^ and CD8^+^ T cell lineages. In B cells instead, *Mzb1* was again found differentially expressed at all the 3 time points. In particular, it was up-regulated in knockout B cells at both baseline and 15 dpi, and down-regulated at 5 dpi.

### Pathway analysis and protein interaction networks

To understand the biological role of Tob1 in the different immune cell subsets, we performed gene ontology (GO) analysis on the nominally significant genes at each EAE time point (Supplementary Tables S2 and S3). At baseline, the most significant enriched category for CD4^+^ T cells was “homophilic cell adhesion” (fold increase: 11.9, P_corr_ = 1.50 × 10^−10^), mainly due to the contribution of the protocadherin alpha (*Pcdha*) and gamma (*Pcdhga*) clusters which are downregulated in Tob1^−/−^ cells. For CD8^+^ T cells instead, the “cellular molecular catabolic process” category was the most enriched at baseline (fold increase: 2.9, P_corr_ = 2.20 × 10^−3^). In particular, several components of the ubiquitin-mediated protein degradation pathway (*Smurf1*, *Ubr4*, *Ubr5*, *Ube2i*, *Ube3a*, *Mid1*, *Usp24*, *Usp35*, *Usp9x*) were down-regulated in Tob1^−/−^ CD8^+^ T cells. Notably, *Smurf1* encodes for an E3 ligase specific for Smad proteins. With regards to B cells, the most significant enrichment was for the category “translation” (fold increase: 8.3, P_corr_ = 12.10 × 10^−14^) due to the up-regulation of several ribosomal protein (*Rpl*) genes in Tob1-knockout B cells. No significant enrichment was identified at 5 dpi for any cytotype. This observation may be related to the fact that even as early as 5 dpi, genes regulated in response to immunization can erase the differences seen at baseline. At 15 dpi, significant categories related to immune response were over-represented in CD4^+^ T cells with the most enriched one being “antigen processing and presentation of peptide antigen via MHC class II” (fold increase: 44.9, P_corr_ = 2.00 × 10^−10^). “Cell cycle process” was instead the most represented category for CD8^+^ T cells at 15 dpi (fold increase: 3.1, P_corr_ = 5.00 × 10^−2^). Also B cells showed an enrichment in ontologies related to cell proliferation with “M phase” as the top category (fold increase: 7.7, P_corr_ = 7.90 × 10^−9^). As expected, several genes encoding cyclins and cyclin-dependent kinases (*Cdk1*, *Ccnb2*, *Ccnf*, *Cdca3*, *Cdca8*) were upregulated in both Tob1-knockout lineages.

**Figure 6:**
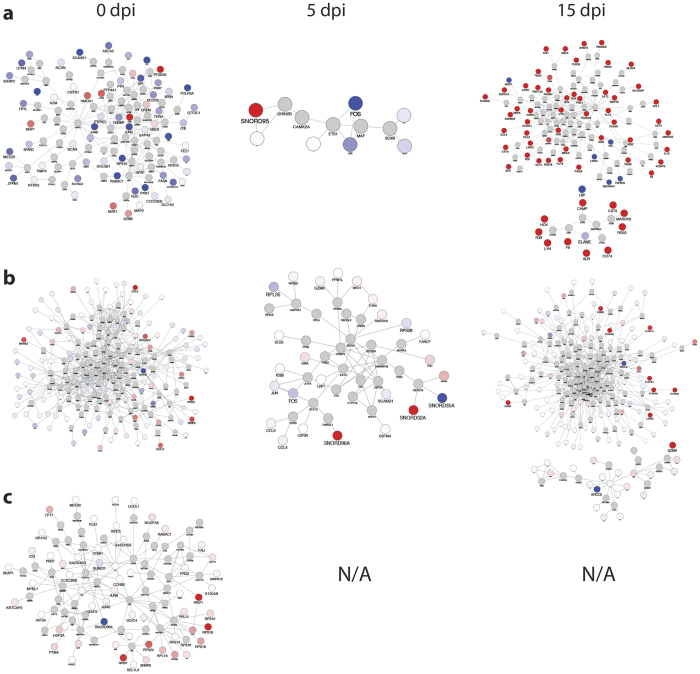
Global interactome analysis identifies biologically relevant gene networks for each immune cell population. (**a**) Significant networks (Z score > 3) enriched in genes with nominal P values from CD4^+^ T cell comparisons (Tob1-KO vs WT) at different EAE stages. One significant network was identified at 0 and 5 dpi while two networks were found at 15 dpi. The analysis was performed using Cytoscape and jActive module plugin. (**b**) Significant networks for CD8^+^ T cells. Similar to CD4^+^ T cells, also CD8^+^ T cells are defined by one network at 0 and 5 dpi and two networks at 15 dpi. (**c**) Significant networks for B cells. Only one network was identified at baseline while no network passed the cutoff for the other two EAE time points.

Next, we explored Tob1 higher functions by means of a previously validated systems biology approach^13^,^14^. We used the Cytoscape software to visualize protein interaction networks using each list of differentially expressed genes as an input to evaluate the possibility that they act in concert, thus altering key biological pathways. A greedy heuristic algorithm was employed to search for all the possible networks from a curated database containing more than 400,000 interactions among 25,000 proteins. All the networks enriched in the input genes with a global score (Z-score) higher than 3 were considered significant. A total of 9 networks were identified in the analysis (Fig. 6a–c and Supplementary Table S4). With the exception of the B cell lineage, which is represented only by one network for baseline, both CD4^+^ and CD8^+^ T cell populations are described by one network at baseline and 5 dpi, and 2 networks at 15 dpi. We then performed GO analysis on each network to look for possible emerging functions that were not captured by the seed genes only. The most represented biological processes among all the networks were “cell cycle regulation” and “transcription”, and given the known function of Tob1 in these processes, this confirms that our approach was able to identify biologically meaningful interaction modules. Remarkably, both networks for CD4^+^ and CD8^+^ T cells at 5 dpi –a time point that failed to show significantly enriched ontologies in our previous analysis– showed an enrichment in the category “regulation of transcription from RNA polymerase II promoter”, further supporting the transcriptional role of Tob1. This finding also highlights the higher sensitivity of the systems approach in detecting biologically meaningful relationships from a large data ensemble. A significant enrichment in “regulation of cell cycle” category connected to transcriptional activity and “SMAD binding” terms was also identified in one of the two networks for CD8^+^ T cells at 15 dpi (fold increase: 7.3, P_corr_ = 2.50 × 10^−18^). Also noteworthy, one of the two networks for CD4^+^ T cells at 15 dpi showed “intracellular signaling cascade” (fold increase: 3.90, P_corr_ = 5.00 × 10^−12^) and “protein amino acid phosphorylation” (fold increase: 5.50, P_corr_ = 5.20 × 10^−12^) as the top categories due to the presence of several genes encoding for protein kinases such as Janus kinase 2 (*Jak2*) and 3 (*Jak3*), protein kinase C alpha (*Prkca*) and spleen tyrosine kinase (*Syk*). A comprehensive list of the pathway analysis results for all the networks is presented in Supplementary Table S5.

### Identification of novel transcripts

**Figure 7:**
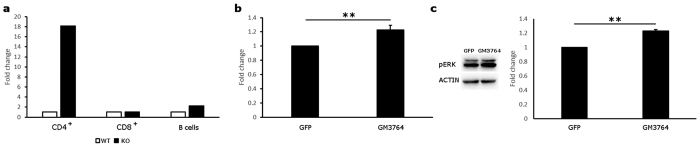
The long non-coding RNA GM3764 is dramatically upregulated in Tob1-KO CD4^+^ T cells and promotes cell proliferation. (**a**) The expression levels for the lncRNA GM3764 were analyzed by quantitative RT-PCR in CD4^+^, CD8^+^ and B cells at baseline (0 dpi). The data were normalized to GAPDH levels which served as internal control. A 16-fold increase in GM3764 expression was detected in Tob1-KO CD4^+^ T cells while a 2-fold increase was identified in KO B cells. No difference was instead reported in CD8^+^ T cells. All experiments were carried out in triplicate and the average values are plotted. (**b**) GM3764 or GFP were overexpressed in 3T3 cells and, after 48 hours, cell growth was assessed by XTT assay. A statistically significant increase in cell proliferation was measured in lncRNA-expressing cells compared to control. Results are presented as mean ± SE and derive from 4 independent transfections. (**c**) Phospho-ERK1/2 levels were probed in GM3764 overexpressing 3T3 cells for 48 hours. A statistically significant increase in phospho-ERK1/2 levels was detected in lncRNA-overexpressing cells compared to control. Endogenous actin levels served as internal loading control. Results are presented as mean ± SE and derive from 3 independent transfections. **P ≤ 0.01, one-tailed T test.

Next generation sequencing technology does not rely on pre-established probes to quantify gene expression but identifies all the actual RNA molecules in a given sample, even those not yet annotated. Thus, we analyzed all the differentially expressed transcripts between wild type and Tob1^−/−^ mice that were not associated to an official gene symbol, looking for possible novel genes regulated by Tob1. At each time point, several candidates defined only by their genomic positions were identified. After manual inspection, the large majority resulted being composed of Ig genes, pseudogenes and endogenous retroviral sequences (SINE and LINE). However, among them we were able to identify a transcript differentially expressed in CD4^+^ T cells at baseline, corresponding to an intergenic long non-coding RNA (lncRNA). According to the ENCODE database, this transcript (id: GM3764) maps to mouse chromosome 3 (88,149,275–88,309,108) and exists in different isoforms due to alternative splicing. The isoform that we found differentially expressed in CD4^+^ T cells is the shortest one, composed of two exons out of nine. We further validated this finding by qRT-PCR, showing that in Tob1-knockout CD4^+^ T cells there was >10 fold increase in the lncRNA levels compared to wild type cells (Fig. 7a). A two-fold increase was also highlighted in Tob1-knockout B cells while no difference was found in CD8^+^ T cells.

We then investigated whether increased levels of GM3764 had a measurable effect on cell function. In particular, given the anti-proliferative role of Tob1, we tested the hypothesis that GM3764 could promote cell division. Thus, GM3764 or GFP were overexpressed in mouse 3T3 cells and then cell growth was assessed by XTT assay after 48 hours from transfection. We were able to measure a statistically significant 20% increase in proliferation (one-tailed T-test; P = 0.019) for cells overexpressing the lncRNA, compared to GFP-expressing control cells (Fig. 7b). Notably, we could detect a concomitant 20% increase in phospho-ERK1/2 levels (one-tailed T-test; P = 0.005) upon GM3764 overexpression (Fig. 7c). Considering the well-established role of this MAP kinase in regulating cell proliferation, the effects of GM3764 on cell growth may, at least in part, be exerted via this molecular pathway.

## Discussion

Tob1 is a negative regulator of the immune response with implications in health and disease. However, little is known about the genetic networks through which Tob1 exerts its functions. In this work, we used a genetic knockout approach and the EAE paradigm to perturb the physiology of the immune system. We then employed the latest “omic” tools to identify changes in gene expression induced by Tob1 ablation in resting condition and upon disease, at both pre- and post-symptomatic stages. We opted for next-generation sequencing instead of microarrays due to better dynamic range of signal quantification as well as the possibility to discover novel transcripts^15^. We show that most of the genes regulated by Tob1 in three main immune cellular subsets –CD4^+^ T cells, CD8^+^ T cells and B cells– are cell-specific. Indeed, our initial approach of testing differential expression in unsorted splenocytes failed to highlight any significant molecular pathway by GO analysis. Our investigation was successful only after isolating the three cell sub-populations. Interestingly, we were able to detect enrichment in specific GO categories at baseline and at the EAE peak, but not at 5 dpi. While the significance of this finding is unclear, we speculate that in the initial stages of disease the extent of the immune response driven by antigen-presentation of MOG peptide was strong enough to overwhelm all the possible differences in terms of gene expression due to Tob1 deficiency. Thus, no specific molecular pathway emerged at 5 dpi by comparing the genetic signatures of knockout and wild type cells. As EAE progresses, the effects of Tob1 deficiency started increasing and tangible differences in specific genetic programs were seen again by day 15 after immunization.

Notably, pathway analysis of genetic profiles at baseline did not show any enrichment in categories directly connected to cell proliferation for any of the three cell subsets analyzed. However, we found that the absence of *Tob1* had an anabolic effect on the physiology of CD8^+^ T cells and B cells, described by the increase in protein translation and the decrease in protein degradation. These cellular processes may still be related to cell cycle as they could represent the necessary steps to prepare the cells for mitosis. In CD4^+^ T cells instead, the only enrichment we found was related to the downregulation of protocadherins in Tob1-knockout mice. These adhesion proteins are predominantly expressed in the nervous system where they mediate diverse neurodevelopmental processes^16^. Nevertheless, an immunological function has been recently identified for at least one member in the subfamily. Indeed, protocadherin-18 was shown to drive differentiation of CD8^+^ T cells to the effector memory phenotype^17^. Thus, it should not be excluded that protocadherins might have a role in the CD4^+^ lineage as well.

Upregulation of genes directly involved in mitosis was seen only at the peak of disease for CD8^+^ T cells and B cells but not for CD4^+^ T cells, which showed instead an enrichment in genes mediating the immune response. However, protein network analysis identified a module for CD4^+^ T cells at 15 dpi significantly enriched in members of the JAK/STAT pathway, a signaling cascade involved in T cell proliferation that is activated by the known Tob1 target interleukin-2 (*Il2*)^5^,^18^. Surprisingly, *Il2* was not part of the list of nominally significant genes for any cell type in our analysis. Nonetheless, a small but consistent increase in *Il2* expression was detected in both Tob1-knockout T cell lineages at baseline and 15 dpi. The generally low level expression of cytokines, and the stringency of our statistical analysis might explain this result.

Importantly, we identified *Mzb1* as a novel Tob1 target that was significantly upregulated in knockout animals before and after EAE in all the three cell types, despite the minimal overlap in their genetic signatures. *Mzb1* encodes for an endoplasmic reticulum calcium regulator and its low levels were shown to induce the arrest of T cell development at pre-TCR selection stage and during positive selection, resulting in peripheral T cell lymphopenia^19^. Moreover, *Mzb1* takes also part in the humoral immune response by enabling proper assembly and secretion of mature immunoglobulins^20^. Hence, Tob1 might control these processes via regulating *Mzb1* expression. Especially upon an autoimmune attack, Tob1-dependent *Mzb1* upregulation might concur to the pathological phenotype by inducing the maturation of encephalitogenic T cells as well as by facilitating the production of auto-reactive antibodies. In addition, the fact that *Mzb1* was found down-regulated in Tob1-knockout B cells at 5 dpi suggests that this gene might be also subjected –given its important role in antibody maturation– to some sort of dynamic control through a negative feedback loop. Such mechanism could be possibly lost as the disease evolves and the immune response overwhelms all the cellular checkpoints. For these reasons, *Mzb1* could represent a novel druggable target in the context of autoimmunity that warrants further study.

Our choice of next generation sequencing also allowed us to discover a novel lncRNA which was dramatically up-regulated in Tob1^−/−^ CD4^+^ T cells at baseline. Emerging evidence has revealed that lncRNAs play a key role in the regulation of several immunological functions including innate and adaptive immune responses and immune cell development^21^. An increasing number of lncRNAs have been found to be involved in autoimmune diseases as well. In the context of MS, the lncRNA *Tmevpg1* seems to be the most reliable candidate for its role in controlling the persistence of Theiler’s murine encephalomyelitis virus (TMEV) infection, an experimental model of MS^22^. In addition, other lncRNAs controlling CD8^+^ T cell differentiation and activation may be involved as well^23^. In our case, the pathophysiological role of the identified lncRNA seems to be connected to cell growth stimulation as suggested by our *in vitro* experiments. This is in agreement with our previous work. In fact, low levels of Tob1 upon disease may promote an aberrant immune response also through GM3764 derepression. Additional experiments in animal models will be required to confirm this preliminary data. Furthermore, the recent evidence that this lncRNA is the top enriched candidate in astrocytes^24^ suggests that might have a role in the nervous system as well.

In conclusion, we have shown for the first time that *Tob1* drives distinct genetic programs in different immune cell populations, although its effects on cell proliferation are phenotypically similar. More importantly, considering the role of *Tob1* in disease progression, the pathways and genes that we have highlighted in this study may represent promising targets to develop novel therapeutic approaches for MS.

## Materials and Methods

### Mouse strains

Tob1^−/−^ mice on a C57BL/6 background were obtained from the RIKEN Bioresource Center. These mice are derived from a line produced and maintained by T. Yamamoto (University of Tokyo, Tokyo, Japan)^25^. Genotyping was performed as previously described^26^. C57BL/6 mice were purchased from The Jackson Laboratory. Tob1^−/−^ and wild type littermates (Tob1^+/+^, C57BL/6) were obtained from a heterozygous breeding of Tob1^+/−^ mice. All animal procedures were performed in compliance with experimental guidelines approved by the University of California, San Francisco committee on animal research (CAR).

### Cell lines

NIH 3T3 cells were obtained from ATCC and maintained in Dulbecco’s Modified Eagle’s medium (GIBCO/Invitrogen) supplemented with 10% v/v fetal bovine serum (GIBCO/Invitrogen) and antibiotics (100 IU/ml penicillin and 100 mg/ml streptomycin) at 37 °C in a humidified atmosphere with 5% CO_2_.

### EAE induction

8 week old Tob1^−/−^ mice and wild type littermates were injected subcutaneously with 100 μg MOG_35–55_ (EZBiolab), in complete Freund’s adjuvant (DIFCO Laboratories). Mice received 400 ng of pertussis toxin intraperitoneally both immediately after immunization and 2 days later. Control mice were injected with everything except the MOG peptide. For all experiments animals were observed daily, and clinical signs were assessed as follows: 0, no signs; 1, decreased tail tone; 2, mild monoparesis or paraparesis; 3, severe paraparesis; 4, paraplegia; 5, quadraparesis; and 6, moribund or death. Mice with an EAE score ≥3 at 15 dpi were used for the experiments. All animal experiments were conducted according to protocols approved by the local animal welfare committee.

### Microarray analysis

Total RNA was extracted using the RNeasy Mini Kit (Invitrogen) from the spleens of Tob1^−/−^ and wild type littermates (3 mice per genotype) both before and 15 days after EAE induction (15 dpi). Excess DNA was digested on columns using the RNase-free DNase Set (Qiagen). RNA quality control, labeling and hybridization onto GeneChip Mouse Gene 1.0 ST Arrays (Affymetrix) were performed at the core facility of the Gladstone Institutes (San Francisco, CA). Microarray data were processed in R using the Bioconductor package and further statistical analyses were carried out using BRB-array Tools (Biometrics Research Branch, NIH). Genes were considered nominally significant if the univariate P-value was less than 0.01. Genes were excluded from the analysis if less than 10% of the expression data showed at least 1.2-fold change in either direction from gene’s median value. Genes with more than 50% of data missing or filtered out were also excluded.

### Cell isolation and RNA-seq

At established time points after EAE induction (0, 5 and 15 dpi) 5 mice per genotype were pooled and their spleens harvested. CD4^+^ T cells, CD8^+^ T cells and B cells were isolated from single cell suspensions by positive immunomagnetic selection using Dynabeads (Invitrogen), following the manufacturer’s instructions. Total RNA was extracted using the RNeasy Mini Kit (Invitrogen), adding lysis buffer directly on the bead-bound cells. DNA was digested on columns using the RNase-free DNase Set (Qiagen). The quality and quantity of RNA was checked with the BioAnalyzer 2100 (Agilent) and all samples had a RIN between 9.1 and 10. Sequencing was performed on an Illumina GAIIx platform with 100-base paired-end reads, following the stranded Truseq protocol. Library preparation and all sequencing steps were performed by Expression Analysis, Inc (Durham, NC). Data analysis was performed using a pipeline implemented into the web-based Galaxy platform^27^,^28^,^29^. In particular, reads were aligned to the most recent mouse genome reference (mm10) and assembled into transcripts using TopHat and Cufflink tools. Cuffdiff was then used to calculate the fragments per kilobase of exon per million fragments mapped (FPKM) score relative to each gene and estimate the differentially expressed genes between genotypes at each selected time point. P values of 0.05 or less were considered significant. Hierarchical clustering was performed with the MultiExperiment Viewer (MeV) software, using the Spearman Rank correlation as distance metric^30^. Principal component analysis (PCA) was performed using the ClustVis web tool^31^.

### Western blot assays

Spleen and brain were dissected and homogenized in 10 volumes of cold lysis buffer (Tris-HCl, pH 6.8, 50 mM; SDS 2%; glycerol 10%, protease inhibitors). Homogenates were then clarified by brief sonication and spun for 5 min at 16000xg to remove cell debris. Supernatants were collected and total proteins were quantified using the Bicinchoninic (BCA) Protein Assay Kit (Pierce). About 20 μg of total proteins were separated on a 10% polyacrylamide gel by SDS-PAGE. Proteins were then transferred to nitrocellulose membranes (Immobilion) at 100 V for 30 min. Membranes were then blocked with 5% milk in Tris buffered saline supplemented with 0.05% Tween-20 (TBS-T) for 1 hour at room temperature (RT). After blocking, membranes were incubated with a primary antibody specific for TOB1 (Sigma) or actin (Cell Signal Technology) in blocking solution (1:1000) overnight at 4 °C. The day after, the membranes were washed 3 times with TBS-T and incubated with horseradish peroxidase (HRP)-conjugated anti-rabbit IgG (Cell Signal Technology) in blocking solution (1:5000) for 1 hour at RT. After extensive washing, membranes were incubated with Supersignal West Dura reagent (Thermo Scientific) and the chemiluminescent signals were detected using a Molecular Imager ChemiDoc XRS System equipped with Quantity One software (Bio-Rad).

### Quantitative RT-PCR analysis

For mRNA analysis, 500 ng of total RNA was retro-transcribed into cDNA with the SuperScript III First-Strand kit (Invitrogen), and 1 μl of the total reaction volume was used for quantitative RT-PCR. All amplifications were performed on a Mx3005P thermocycler (Stratagene), using the Power SYBR Green PCR Master Mix (Applied Biosystem). Gene expression levels were analyzed using the following primers: CD4 forward, 5′-TCCTTCCCACTCAACTTTGC-3′; CD4 reverse, 5′-AAGCGAGACCTGGGGTATCT-3′; CD8 forward, 5′-GCTCAGTCATCAGCAACTCG-3′; CD8 reverse, 5′-ATCACAGGCGAAGTCCAATC-3′; B220 forward, 5′-CAAAGTGACCCCTTACCTGCT-3′; B220 reverse, 5′-CTGACATTGGAGGTGTGTGT-3′; GM3764 forward, 5′-AAGCCTCATCCTGACATCCT-3′; GM3764 reverse, 5′-TTGGTCTGTGGTCCAGAAAG-3′; GAPDH forward, 5′-CATGGCCTTCCGTGTTCCTA-3′; GAPDH reverse, 5′-CCTGCTTCACCACCTTCTTGAT-3′. Each sample was run in triplicate and the ΔΔC_t_ method was used for relative quantification, with GAPDH expression levels serving as internal control.

### Cell proliferation analysis using FACS

Splenocytes were isolated from the spleens of 8–12 week old Tob1^−/−^ mice and wild type littermates. After loading with eFluor-670 proliferation dye (eBioscience #65-0840-85) according to the instructions, splenocytes were seeded into 96-well U-bottom plates at the concentration of 10^6^ cells/mL in RPMI 1640 supplemented with 2 mM L-glutamine, 1 mM sodium pyruvate, 100 IU/mL penicillin, 100 mg/mL streptomycin, 50 μM 2-mercaptoethanol and 10% fetal bovine serum (FBS) and stimulated with 5 μg/mL lipopolysaccharides (LPS) from E. Coli 0111:B4 (Sigma) or left unstimulated as a control for 3 days. After stimulation, cells were incubated in 10% normal mouse serum (Jackson ImmunoResearch #015-000-120) and 1% anti-mouse CD16/CD32 antibody (BD Pharmingen #553141) in flow cytometry staining buffer (0.5% BSA, 2 mM EDTA in PBS) to reduce nonspecific binding, labeled with live/dead cell marker (Live/Dead Fixable Aqua Dead Cell Stain Kit, Thermo Fisher #L-34957) and immunostained with a PE-conjugated anti-B220 antibody (BD Pharmingen #553089) using standard flow cytometry staining techniques. After fixing in 2% paraformaldehyde in PBS, the cells were subjected to flow cytometry analysis using LSR II analyzer (BD Biosciences). Flow cytometry data were analyzed using FlowJo software by gating for lymphocytes based on forward/side scatter, followed by singlet selection and dead cell exclusion, followed by gating for B220-PE expression to select B cell population. All gating was based on fluorescence-minus-one controls.

### Cytokine and IgG quantification

B cells were isolated from the spleens of 7–9 week old Tob1^−/−^ mice and wild type littermates by negative selection, using Dynabeads Mouse CD43 (Untouched B cells) (Invitrogen). Purified B cells were then seeded into 24-well plates at the concentration of 0.5 × 10^6^/mL in complete RPMI 1640 medium and stimulated with 5 μg/mL LPS or left unstimulated as a control for 3 days. Conditioned media were subsequently probed by ELISA for the levels of cytokines IL-6 and IL-10 and immunoglobulins IgG and IgM. The Mouse IL-6 and IL-10 ELISA Kits (Millipore) and Mouse IgG and IgM ELISA kits (Abcam) were used according to the manufacturers’ instructions.

### Pathway analysis

The cellular component, molecular function and biological process ontologies were annotated for each set of differentially expressed genes (nominally significant, uncorrected P < 0.05), using the DAVID tool^32^,^33^. Enriched terms in each gene ontology (GO) category were searched against the whole mouse transcriptome, adopting FAT filters. Benjamini–Hochberg post-hoc test was used for multiple-test correction and significance was considered at corrected P values of 0.05 or less.

### Global interactome analysis

Protein interaction networks were generated using the Cytoscape software^34^. The lists of nominally significant genes from each pairwise comparison were used as input and the neighbors of each node were added from a high-quality curated protein–protein interaction network accessible at the Human Protein Reference Database (HPRD; http://www.hprd.org/). Cytoscape plugin jActives modules was then used to calculate a global score (Z-score) for all the possible networks that could be generated from each input gene list using the following parameters: 1000 for the maximum number of modules, 0.2 for the overlap threshold between modules and 2 for the search depth. Networks with Z-scores > 3.0 were considered significant.

### DNA constructs

The sequence of the lncRNA GM3764 was amplified from CD4^+^ T cell cDNA using the Phusion High Fidelity PCR kit (New England Biolabs) with the following primers: Forward, 5′-CCCCCCCTCGAGAAGCTTGGTACAGGAACTTCG-3′; Reverse, 5′-CCCCCCTCTAGACACAGTATGCAATGTTCTTTATT-3′. The PCR product was then double-digested with the restriction enzymes XhoI and XbaI (New England Biolabs) and cloned into the pcDNA3 vector (13031; Addgene) from which the coding sequence of GFP had been previously excised using the same pair of enzymes. Cloning was confirmed by Sanger sequencing.

### Cell proliferation assays

NIH 3T3 cells were grown in 6-well plates and transfected at 90% confluence with pcDNA3 plasmid (expressing GM3764 or GFP) using Lipofectamine LTX (Invitrogen), according to manufacturer’s instructions. After 24 hours, transfected cells were re-seeded into 96-well plates at a concentration of 5,000 cells/well and let grow for additional 24 hours. The day after, cell proliferation was assessed with the Cell Proliferation Kit II (Roche), incubating cells with the XTT labeling mix for 4 hours. Absorbance was subsequently read at 492 nm with reference wavelength at 690 nm, using a VersaMax plate reader (Molecular Devices).

### ERK activation analysis

NIH 3T3 cells were transfected with pcDNA3 plasmid (expressing GM3764 or GFP) and after 48 hours were lysed in RIPA buffer supplemented with protease and phosphatase inhibitors (Roche). Cell extracts were then clarified by centrifugation at 2,300xg for 5 min. An amount equal to 10–20 μg of total proteins was separated by 10% SDS-PAGE and ERK1/2 activation was assessed by Western blot as previously described, using a monoclonal antibody specific for phospho-p44/42 Map Kinase (Thr202/Tyr204) (Cell Signaling). Actin levels were used as loading control.

## Additional Information

**How to cite this article**: Didonna, A. *et al*. Immune cell-specific transcriptional profiling highlights distinct molecular pathways controlled by Tob1 upon experimental autoimmune encephalomyelitis. *Sci. Rep*. **6**, 31603; doi: 10.1038/srep31603 (2016).

## Supplementary Material

Supplementary Information

Supplementary Data S1

Supplementary Data S2

Supplementary Data S3

Supplementary Data S4

Supplementary Data S5
