# Supplementary material for: Immune cell-specific transcriptional profiling highlights distinct molecular pathways controlled by Tob1 upon experimental autoimmune encephalomyelitis
Source: Sci Rep. 2016 Aug 22;6:31603. doi: 10.1038/srep31603 (PMC4992865; doi:10.1038/srep31603)
Supplement: Supplementary Information [file srep31603-s1.pdf]

Supplementary Material for the manuscript:

Immune cell-specific transcriptional profiling highlights distinct molecular pathways controlled by Tob1 upon experimental autoimmune encephalomyelitis

Alessandro Didonna[1], Egle Cekanaviciute[1], Jorge R. Oksenberg[1], Sergio E. Baranzini[1*]

[1]Department of Neurology, University of California San Francisco, San Francisco, California 94158, USA

***Corresponding author:** Sergio.Baranzini@ucsf.edu

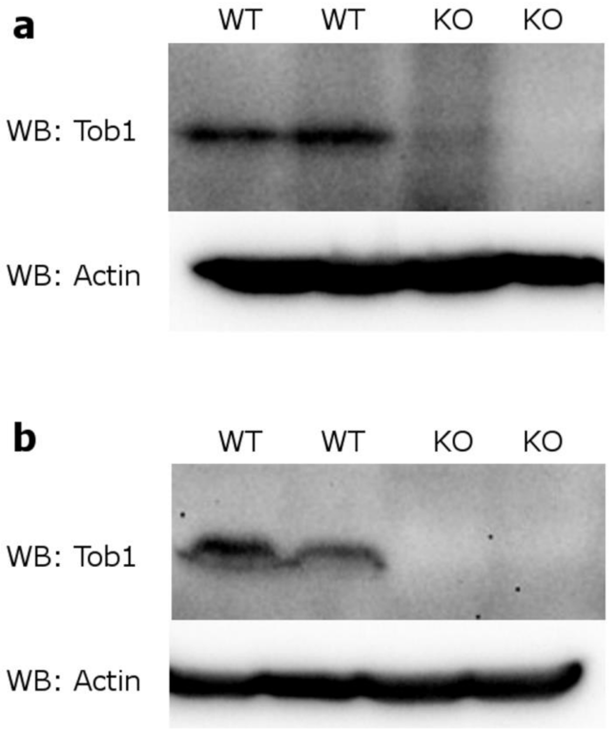

Supplementary Figure S1. Tob1-KO mice do not express Tob1 protein. Spleen and brain tissues from Tob1-KO and WT age-matched controls were tested for Tob1 expression by western blot. About 20 μg of total proteins from brain and spleen lysates were separated by SDS-PAGE and probed with antibodies specific for Tob1 or actin. A positive signal for Tob1 was detected only in WT animals in both tested tissues. Actin staining confirmed that equal amounts of proteins were loaded in each lane. Two animals per genotype were used for this analysis.

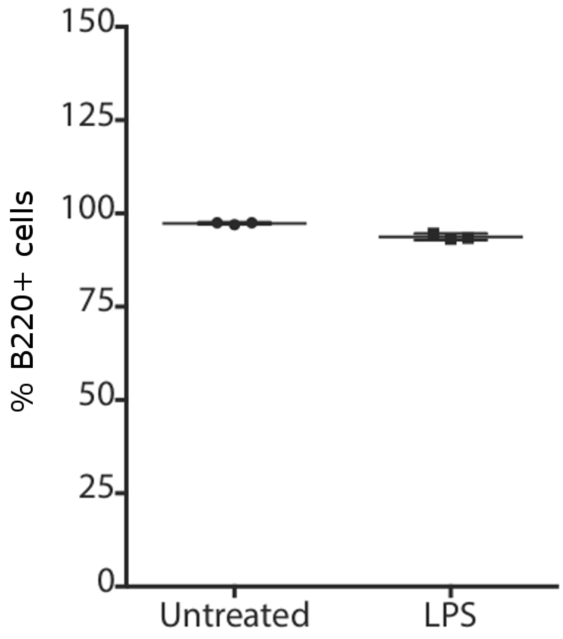

Supplementary Figure S2. Negative selection of B cells reaches high purity. Untouched B cells were isolated by negative selection from the spleen of three mice and stimulated with 5 μg/mL lipopolysaccharides (LPS) for 3 days or left unstimulated. Cells were then fixed and stained with PE-conjugated antibodies specific for CD4, CD8 or B220. A purity close to 100% was detected for the B cell population (B220 positive cells) either before or after LPS stimulation. Results are presented as mean±SE.

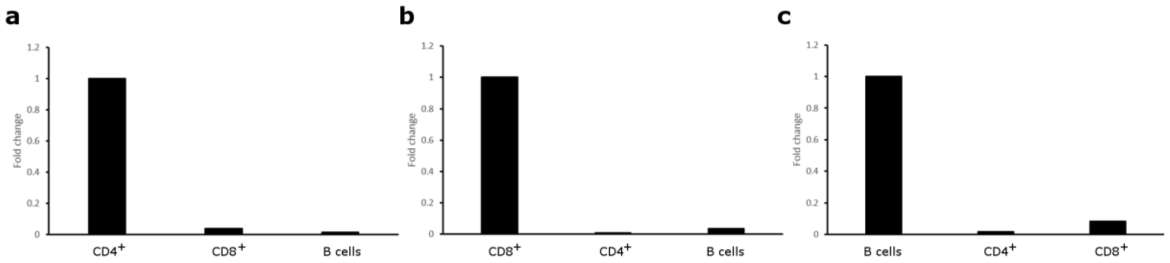

Supplementary Figure S3. Magnetic isolation of immune cell populations reaches high purity. (a) *CD4*, (b) *CD8* and (c) *B220* transcript levels were analyzed by quantitative RT-PCR in CD4$^+$ T cell, CD8$^+$ T cell and B cell populations. The different cell subsets were isolated by magnetic purification from the spleens of WT mice. Data were normalized on *GAPDH* levels which served as internal control. The enrichment for each specific cell population is >95%. All reactions were run in triplicate and the average values are plotted.
